# Peripheral pulmonary nodules: Relationship between multi-slice spiral CT perfusion imaging and tumor angiogenesis and VEGF expression

**DOI:** 10.1186/1471-2407-8-186

**Published:** 2008-06-30

**Authors:** Shu-Hua Ma, Hong-Bo Le, Bao-hui Jia, Zhao-Xin Wang, Zhuang-Wei Xiao, Xiao-Ling Cheng, Wei Mei, Min Wu, Zhi-Guo Hu, Yu-Guang Li

**Affiliations:** 1 Department of Radiology, First Affiliated Hospital, Medical College of Shantou University, Shantou 515041, PR China; 2 Guangdong Key Laboratory of Medical Molecular Imaging, Shantou 515041, PR China; 3 Guang Anmen Hospital, China Traditional Chinese Medical Sciences, Beijing 100053, PR China

## Abstract

**Background:**

The aim of this study is to investigate the relationship between16-slice spiral CT perfusion imaging and tumor angiogenesis and VEGF (vascular endothelial growth factor) expression in patients with benign and malignant pulmonary nodules, and differential diagnosis between benign and malignant pulmonary nodules.

**Methods:**

Sixty-four patients with benign and malignant pulmonary nodules underwent 16-slice spiral CT perfusion imaging. The CT perfusion imaging was analyzed for TDC (time density curve), perfusion parametric maps, and the respective perfusion parameters. Immunohistochemical findings of MVD (microvessel density) measurement and VEGF expression was evaluated.

**Results:**

The shape of the TDC of peripheral lung cancer was similar to those of inflammatory nodule. PH (peak height), PHpm/PHa (peak height ratio of pulmonary nodule to aorta), BF (blood flow), BV (blood volume) value of peripheral lung cancer and inflammatory nodule were not statistically significant (all P > 0.05). Both showed significantly higher PH, PHpm/PHa, BF, BV value than those of benign nodule (all P < 0.05). Peripheral lung cancer showed significantly higher PS (permeability surface) value than that of inflammatory nodule and benign nodule (all P < 0.05). BV, BF, PS, MTT, PH, PHpm/PHa, and MVD among three groups of peripheral lung cancers were not significantly (all P > 0.05). In the case of adenocarcinoma, BV, BF, PS, PHpm/PHa, and MVD between poorly and well differentiation and between poorly and moderately differentiation were statistically significant (all P < 0.05). The peripheral lung cancers with VEGF positive expression showed significantly higher PH, PHpm/PHa, BF, BV, PS, and MVD value than those of the peripheral lung cancer with VEGF negative expression, and than those of benign nodule with VEGF positive expression (all P < 0.05). When investigating VEGF negative expression, it is found that PH, PHpm/PHa, and MVD of inflammatory nodule were significantly higher than those of peripheral lung cancer, PS of inflammatory nodule were significantly lower than that of peripheral lung cancer (all P < 0.05). PH, PHpm/PHa, BF, and BV of benign nodule were significantly lower than those of inflammatory nodule (all P < 0.05), rather than PS and MTT (mean transit time) (all P > 0.05). PH, PHpm/PHa, BV, and PS of benign nodule were significantly lower than those of peripheral lung cancer (all P < 0.05). In the case of VEGF positive expression, MVD was positively correlated with PH, PHpm/PHa, BF, BV, and PS of peripheral lung cancer and PS of benign nodule (all P < 0.05).

**Conclusion:**

Multi-slice spiral CT perfusion imaging closely correlated with tumor angiogenesis and reflected MVD measurement and VEGF expression. It provided not only a non-invasive method of quantitative assessment for blood flow patterns of peripheral pulmonary nodules but also an applicable diagnostic method for peripheral pulmonary nodules.

## Background

Pulmonary nodule is a kind of common disease found in the lung. Sometimes it is difficult to directly identify between malignancy and benignancy of peripheral pulmonary nodule by using morphology features because of changes in the blood vessel volume, perfusion and capillary permeability in new tumor vessels which may result in the changes of blood patterns [1-3]. The method of perfusion imaging is effective to detect the differentiation of blood supply between benign and malignant pulmonary nodules in quantity and quality [4-8].

The purpose of this study was to quantitatively evaluate 16-slice spiral CT perfusion imaging to describe the blood patterns of benign and malignant pulmonary nodules and to investigate the relationship between CT perfusion imaging and tumor angiogenesis and vascular endothelial growth factor (VEGF) expression in patients with benign and malignant pulmonary nodules, and the differential diagnosis between benign and malignant pulmonary nodules by using perfusion imaging of 16-slice spiral CT.

## Methods

### 1. Patients

From January 2005 to December 2006, 69 patients with peripheral pulmonary nodule were studied with informed consent. All the tumors were conformed by surgery. The patients underwent 16-slice spiral CT perfusion scanning in less than a week before surgery. No patients received any therapy (such as chemotherapy) before surgery. Five of 69 patients who could not hold their breath, led to errors in perfusion values and artifacts on parametric maps, and so were excluded. The other 64 patients (39 men and 25 women; age range, 26–75 years; mean age ± SD, 51.27 ± 13.83 years; approximately spherical; long-axis diameters range, 1.6–4.2 cm, mean diameters, 2.8 cm) were included.

The 64 patients included 39 cases of peripheral lung cancer (24 adenocarcinoma, 11 squamous cell carcinoma, 2 large cell carcinoma, and 2 small cell carcinoma, long-axis diameters range, 1.7–4.2 cm, mean diameters, 2.7 cm), 13 cases of inflammatory nodules (8 active inflammatory granuloma and 5 pneumonia apostematosa, long-axis diameters range, 2.1–3.9 cm, mean diameters, 3.2 cm), 12 cases of benign nodules (9 tuberculoma and 3 hamartoma, long-axis diameters range, 1.6–3.0 cm, mean diameters, 2.5 cm). In the 39 cases of peripheral lung cancer, 24 adenocarcinoma (well, moderately, poorly differentiation are 9, 10, 5 cases respectively), 11 squamous cell carcinoma (well, moderately, and poorly differentiation are 6, 4, 1 cases respectively), 11 large and small cell carcinoma (well, moderately, poorly differentiation are 6, 4, 1 cases respectively) (Table 1).

**Table 1:** Differentiation of peripheral lung cancer

Differentiation of lung tumors	Cases	Well	Moderately	Poorly
Adenocarcinoma	24	9	10	5
Squamous cell carcinoma	11	6	4	1
Large and small cell carcinoma	4	3	0	1

The experiments were performed with the approval of the human subjects ethics committee of the First Affiliated Hospital, Medical College of Shantou University.

### 2. Computed tomography scanning

Computed tomography perfusion imaging were performed by using a commercially available 16-slice spiral CT scanner (Lightspeed, GE, USA). The patients were instructed in breath-holding and in holding their breath at full suspended inspiration during quiet breathing so as to facilitate to repeat imaging of the same tissue volume.

The CT scan involved two steps. Firstly, a conventional CT scan was performed to find the tumor and determine the section of perfusion scan. The scan ranged from the thoracic inlet to the pulmonary base, with 1.25-mm section thickness, 1.375 pitch, 10-mm reconstruction thickness, 120-kV tube voltage, 220-mA tube current. Secondly, CT perfusion scan was performed after the tumor was found.

Four adjacent sections containing the largest nodular diameter were selected as the target cross sections. Nonionic contrast medium ultravist 300 (300 mgI/ml)was injected via a superficial antebrachial vein with pressure syringe at a flow rate of 4 ml/s and a total volume of 50 ml. Perfusion scan was in a cine pattern, with 0.5 s/coil, 5-mm section thickness × 4, 0-mm interval, no inclination of scan frame, tube voltage of 120 kV, tube current of 60 mA, exposition time of 40 s, data acquisition time of 40 s, delay time of 2 s (perfusion scan started after injection of contrast medium for 2 s).

### 3. Analysis of CT perfusion images

The 316 images (79 images per section, 4 sections) with 5-mm reconstruction thickness generated by CT perfusion were transmitted to AW4.1 workstation via a local area network. All the data were calculated quantitatively according to the calculation approach of the deconvolution model with the CT body perfusion software of GE perfusion3 and produced perfusion parametric maps (assigned type). The analysis package offered a motion correction for in-plane movement. The four sections of data were respectively analyzed. Air and bone pixels are excluded from calculations by CT value (Hounsfield Unit, HU) thresholds, typically -120 to 240 HU.

Firstly, descending aorta (or innominate artery without descending aorta) in-plane of nodule was chosen as the input artery. A region of interest (ROI) with five pixels was hand drawn in the centre of the descending aorta. Secondly, three ROIs were hand drawn in every section of the nodular area of perfusion parametric maps; every ROI was about 70 pixels; and calcified, cystic, or necrotic areas were avoided as far as possible. The edges of the nodule were avoided to prevent partial volume. We examined an ROI that covered about two-thirds of the diameter of the nodule at the equator if the nodule was smaller.

The time density curves (TDC) of the descending aorta and pulmonary nodule ROI, peak height (PH), peak height ratio of pulmonary nodule to aorta (PHpm/PHa), each type of perfusion parametric maps [blood flow (BF), blood volume (BV), mean transit time (MTT), permeability surface (PS)], and respective perfusion parameters for all four anatomic section locations available for each patient were calculated. Representative parameter values of three groups of pulmonary nodules were then averaged across the four sections.

Two radiologists experienced in chest CT respectively observed the TDCs and perfusion parametric maps.

### 4. Immunohistochemical staining

The position from which the pathologic tissue samples were drawn as far as possibe kept a parallel with the CT perfusion section. The tissue in each case was fixed by formalin and embedded by paraffin. Paraffin sections (5 μm thickness) were used for immunohistochemistry using a streptavidin peroxidase (SP) kit and a hematoxylin and eosin staining kit. The slices were deparaffinized and dehydrated in graded alcohols. Heat-induced antigen retrieval was performed by using a microwave oven and citrate buffer (pH, 6.0; 10 mol/L). All samples were immunostained by using the SP procedure with the monoclonal mouse antibodies VEGF (1:100 dilution) and the monoclonal rabbit antibodies CD34 (1:100 dilution) (both from Fuzhou Maixin Company, China). The positive breast cancer paraffin sections supplied with the SP kit were taken as positive controls and samples with primary antibody replaced by PBS were taken as negative controls.

VEGF positive brown staining is located in the cytoplasm. The VEGF staining was graded in terms of its extent and intensity shown by Mattern et al. [9]. To evaluate the VEGF expression, a score corresponding to the sum of both (a) staining intensity (0 = negative; 1 = weak; 2 = intermediate; 3 = strong) and (b) percentage of positive cells (0 = 0% positive cells;1 ≤ 25% positive cells; 2 = 25–50% positive cells; 3 ≥ 50% positive cells) was established. The sum of (a) and (b) reached a maximum score of 6. We observed 10 fields of vision by high time lens (×400) and calculated the average score: 0–2 is negative, more than 2 is positive, between 3 and 4 is weak positive expression, between 5 and 6 is strong positive expression.

The degree of angiogenesis was determined by means of MVD measurement in the defined areas of tissue sections according to the Weidner method [10]. Each slide was first scanned at low magnification (×100) to determine five "hotspot" areas where the number of microvessels was at a maximum. Calcified or necrotic areas were excluded. MVD was counted in each of the five hotspot areas on a slide at high magnification (×200). Any one brown staining endothelial cell or cell cluster that is obviously different from peripheral tissues and connective tissues was counted as a single vessel, and branch construct with discrete breaks was also counted as a single vessel. Appearance of erythrocyte and lumen of vessel may not be taken as calculation standard for MVD measurement; lumen of vessel that is more than eight erythrocytes in diameter and vessel that has thicker muscular layer were also not taken as calculation standard for MVD measurement.

A pathologist experienced in lung pathology recorded the histologic diagnosis of each pulmonary nodule. MVD measurement and VEGF expression were assessed by means of immunohistochemistry.

### 5. Data and statistical analysis

**Figure 1:**
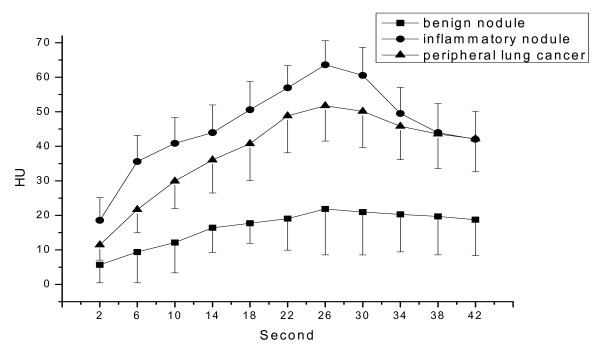
Time density curve of peripheral lung cancer, inflammatory nodule, and benign nodule.

The pulmonary nodules were divided into three groups (peripheral lung cancer, inflammatory nodule, benign nodule). HU values of 11 time points (2, 6, 10, 14, 18, 22, 26, 30, 34, 38, 42 s) on each TDC of ROI at four anatomic section locations available for each group of pulmonary nodules were recorded. Representative HU values of the respective time point were then averaged (Table 2). The TDCs of the three groups of pulmonary nodules were drawn by Graph software (OriginPro 7.5) (Figure 1).

**Table 2:** HU values showing 11 time-points on the TDC of peripheral lung cancer, inflammatory nodule, and benign nodule

Pathologic type	2 S	6 S	10 S	14 S	18 S	22 S	26 S	30 S	34 S	38 S	42 S
Peripheral lung	11.46	21.66	29.94	36.05	40.76	48.86	51.77	50.16	45.81	43.55	42.16
Cancer	±4.41	±6.71	±7.99	±9.54	±10.64	±10.75	±10.21	±10.50	±9.67	±10.02	±9.54
Inflammatory	18.55	35.60	40.86	43.96	50.60	56.97	63.64	60.57	49.52	43.98	42.02
Nodule	±6.60	±7.59	±7.47	±8.05	±8.17	±6.37	±6.97	±8.11	±7.56	±8.41	±8.18
Benign nodule	5.68	9.39	12.14	16.41	17.73	19.08	21.84	20.97	20.28	19.68	18.73
	±5.25	±8.94	±8.82	±7.13	±5.83	±9.16	±13.28	±12.47	±10.84	±11.11	±10.46

*Data is the mean ± SD.

The BV, BF, MTT, PS, PH, PHpm/PHa value of pulmonary nodule ROI for all four anatomic section locations available for each group of patients were recorded. Representative parameter values were then averaged. All statistical results were indicated with mean ± SD.

Chi-square test was used among two or three groups of number datas. P value was calculated by Exact Method. One-way ANOVA was used to analyze statistical differences in two or three groups of measurement data, P value was calculated by LSD Method for equation of variance or by Tamhane Method for heterogeneity of variance. P values less than 0.05 were considered to indicate a statistically significant difference. Pearson coefficients were used to represent the relationship between the perfusion value (BV, BF, MTT, PS, PH, PHpm/PHa) and MVD measurement. All statistical analyses were performed by using a statistical software (SPSS 13.0 for Windows).

## Results

### 1. Time density curve of peripheral pulmonary nodule

**Figure 2:**
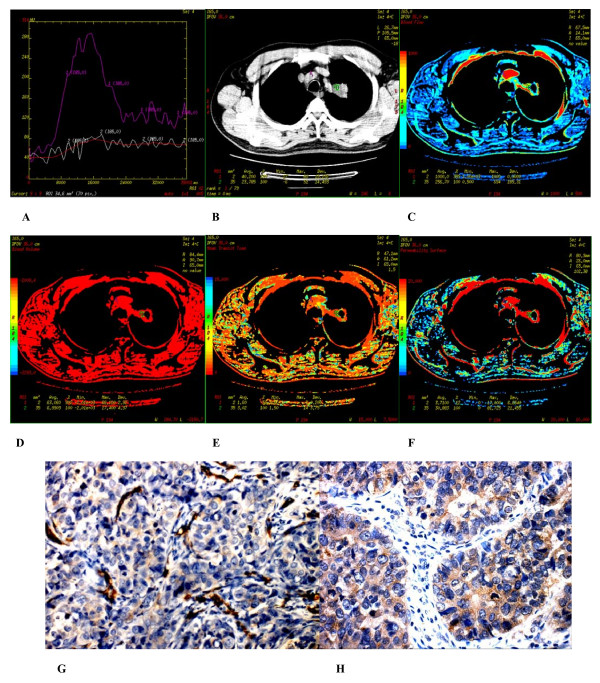
**(A-H) **Poorly differentiated adenocarcinoma found in the apicoposterior segment of superior lobe of the left lung of a 56 year-old male. **(A) **Time density curve. **(B-F) **(original image, BF, BV, MTT, PS) typeI parametric maps, PS value is higher (30.883). **(G) **CD34 staining shows many immature tumor microvessels (× 200). **(H) **VEGF expression is strong positive (× 400).

**Figure 3:**
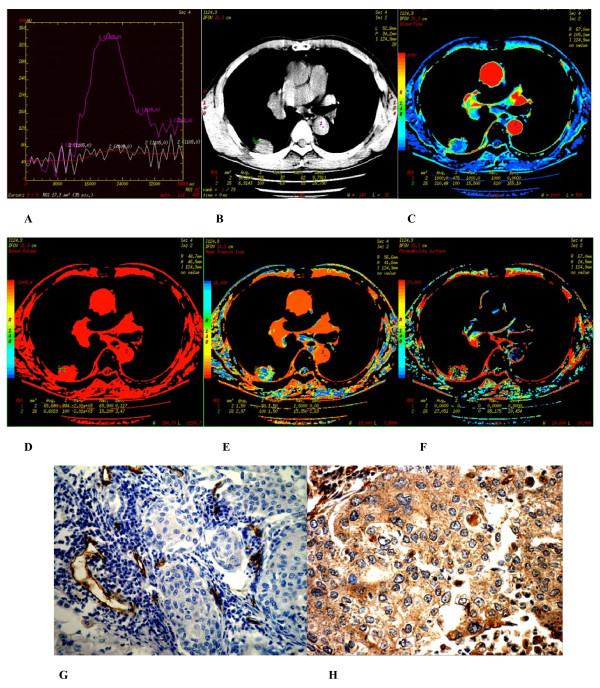
**(A-H) **Well differentiated squamous cell carcinoma found in the posterior basal segment of inferior lobe of the right lung of a 61-year-old male. **(A) **Time density curve. **(B-F) **(original image, BF, BV, MTT, PS) TypeII parametric maps, PS value is higher (27.051). **(G) **CD34 staining shows many immature tumor microvessels (× 200). **(H) **VEGF expression is strong positive (× 400).

**Figure 4:**
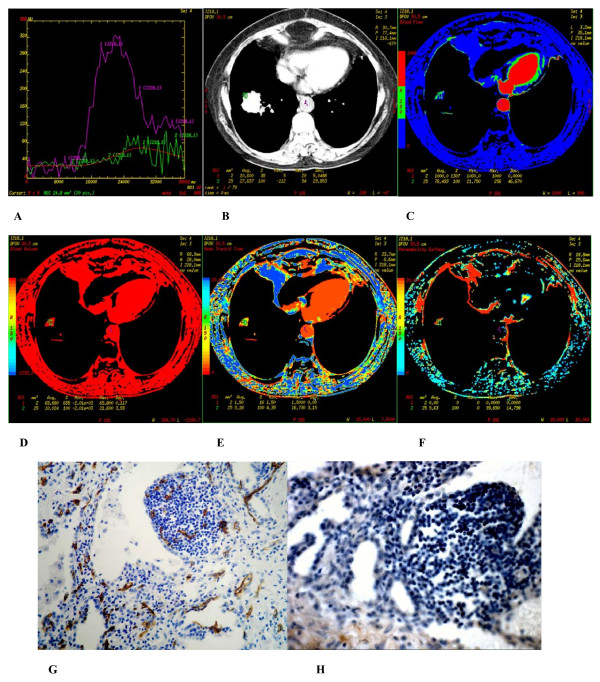
**(A-H) **Inflammatory nodule found in segmentum basale anterius of inferior lobe of the right lung of a 46-year-old male. **(A) **Time density curve. **(B-F) **(original image, BF, BV, MTT, PS) TypeIII parametric maps, PS value was lower (5.63). **(G) **CD34 staining shows many expanded mature microvessels (× 200). **(H) **VEGF expression is negative (× 400).

**Figure 5:**
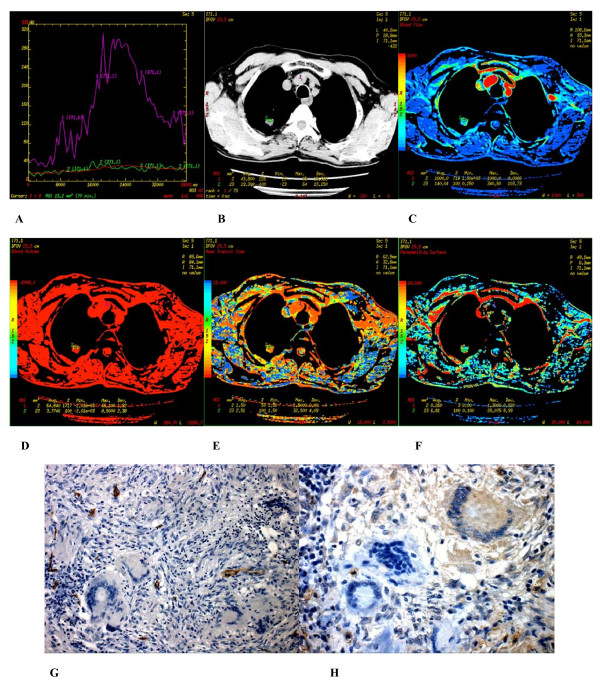
**(A-H) **Tuberculoma found in apical segment of superior lobe of the right lung of a 39-year-old female. **(A) **Time density curve. **(B-F) **(original image, BF, BV, MTT, PS) typeI parametric maps, PS value was lower (6.81). **(G) **CD34 staining shows a few of more mature microvessels (× 200). **(H) **VEGF expression is negative (× 400).

**Figure 6:**
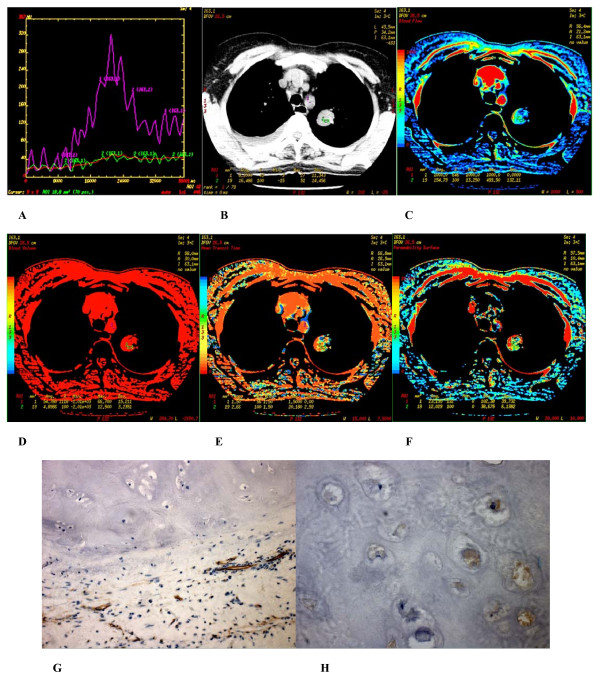
**(A-H) **Hamartoma found in apicoposterior segment of superior of the left lung of a 54-year-old male. **(A) **Time density curve. **(B-F) **(original image, BF, BV, MTT, PS) type I parametric maps, PS value was moderate (12.029). **(G) **CD34 staining shows a few of more mature microvessels (× 200). **(H) **VEGF expression is negative (× 400).

The shape of the TDC of peripheral lung cancer was similar to that of inflammatory nodule. Both showed a steeper slope, and had an obviously increased nodular HU value. However, TDC of peripheral lung cancer changed little after reaching a peak. It became flat at the peak, and had a platform. However, TDC of inflammatory nodule declined immediately after reached peak and did not have a platform. Moreover, inflammatory nodule showed a higher peak than that of peripheral lung cancer. TDC of both was obviously different from that of benign nodule, in which, the TDC changed little, had no steeper slope, had a flat trend, and had no obvious increase in nodular HU value (Figures 1, 2A–6A).

### 2. Immunohistochemistry

In peripheral lung cancers, positive staining of CD34 were found in the tissues of small artery, small vein, capillary and blood vessel endothelium of lung cancer tissues, which were stained brown. Microvascular distributions were intense in the surrounding and interstitial substances of the nodules, and blood vessels were rare in essence and necrotic areas. Many immature tumor capillaries were found in the interstitial substance of the nodules. Many single or cluster endothelial cell clumps showed no lumina or their lumina were closed (Figures 2G–3G). In peripheral lung cancers, VEGF positive staining was located in the cytoplasm. The size of the cancer cell nucleus was bigger and atypia of the nucleus was obvious (Figures 2H, 3H). VEGF expression was found positive in 29 patients and negative in 10 patients.

In inflammatory nodules, positive staining of CD34 which displayed more expanded mature blood capillaries were observed (Figure. 4G). VEGF expression in all the 13 cases with inflammatory nodule were negative (Figure 4H).

In benign nodules, positive staining of CD34 manifested that a smaller quantity of staining tumor capillaries were observed (Figures 5G, 6G). For VEGF expression, 3 patients were positive, 9 patients were negative (Figures 5H, 6H).

MVD results of peripheral lung cancer, inflammatory nodule and benign nodule were 29.292 ± 14.391, 33.966 ± 15.548, and 7.959 ± 2.328, respectively. MVD values of peripheral lung cancer and inflammatory nodule were very high and not statistically significant (P > .05). However, both MVD values were statistically significant higher than that of benign nodule (all P < .05) (Tables 3, 4).

**Table 3:** Perfusion parameters and MVD of three groups of pulmonary nodules

Parameter	Peripheral lung cancer (n = 39)	Inflammatory nodule (n = 13)	Benign nodule (n = 12)
BV	10.577 ± 3.708	8.542 ± 2.581	5.806 ± 1.628
BF	205.232 ± 90.669	160.902 ± 68.412	80.733 ± 16.060
MTT	7.472 ± 3.760	6.397 ± 3.292	5.760 ± 2.884
PS	19.703 ± 7.660	4.019 ± 1.730	3.797 ± 2.186
PH	60.790 ± 8.336	65.700 ± 5.948	27.833 ± 9.072
PHpm/PHa (100%)	20.611 ± 3.243	22.163 ± 2.240	8.893 ± 2.624
MVD	29.292 ± 14.391	33.966 ± 15.548	7.959 ± 2.328

*Data is the mean ± SD.

**Table 4:** Comparison among the perfusion parameters and MVD of three groups of pulmonary nodules

Parameter	BV (p)	BF (p)	MTT (p)	PS (p)	PH (p)	PHpm/PHa (p)	MVD (p)
Peripheral lung cancer (n = 39) and inflammatory nodule(n = 13)	0.106	0.207	0.345	0.000	0.062	0.107	0.726
Peripheral lung cancer (n = 39) and benign nodule(n = 12)	0.000	0.000	0.146	0.000	0.000	0.000	0.000
Inflammatory nodule(n = 13) and benign nodule(n = 12)	0.013	0.003	0.653	0.990	0.000	0.000	0.000

MVD among adenocarcinoma, squamous cell carcinoma, large and small cell carcinoma were not significantly (all P > 0.05) (Table 5). In the case of adenocarcinoma, MVD between poorly and well differentiation and between poorly and moderately differentiation were statistically significant (all P < 0.05) (Tables 6, 7).

**Table 5:** Comparison among the perfusion parameters and MVD of adenocarcinoma, squamous cell carcinoma, large and small cell carcinoma

Parameter	Cases	BV	BF	MTT	PS	PH	PHpm/PHa	MVD
Adenocarcinoma	24	10.173 ± 3.624	225.46 ± 83.132	6.673 ± 3.821	17.819 ± 8.214	62.329 ± 8.084	21.159 ± 3.083	27.483 ± 14.85
Squamous cell carcinoma	11	10.244 ± 3.528	164.071 ± 101.015	8.876 ± 3.371	22.217 ± 6.414	56.436 ± 8.456	19.048 ± 3.465	30.828 ± 15.559
Large and small cell carcinoma	4	13.92 ± 3.925	197.063 ± 86.943	8.413 ± 3.969	24.093 ± 3.389	63.525 ± 6.52	21.62 ± 2.758	35.918 ± 5.683

*BV, BF, PS, MTT, PH, PHpm/PHa, and MVD among adenocarcinoma, squamous cell carcinoma, large and small cell carcinoma were not significantly (all P > 0.05).

**Table 6:** Tumor differentiations of adenocarcinoma, squamous cell carcinoma, large and small cell carcinoma

Parameter	Differentiation	Cases	BV	BF	MTT	PS	PH	PHpm/PHa	MVD
Adenocarcino ma (24 cases)	well	9	8.706 ± 2.441	205.723 ± 77.469	5.422 ± 1.591	15.949 ± 5.736	61.122 ± 6.371	20.718 ± 3.001	22.451 ± 13.536
	moderately	10	9.261 ± 3.448	205.193 ± 90.827	8.755 ± 5.0	15.528 ± 9.547	59.95 ± 6.435	20.069 ± 2.478	22.831 ± 12.514
	poorly	5	14.636 ± 2.237	301.518 ± 6.290	4.758 ± 1.919	25.766 ± 4.142	69.26 ± 11.132	24.134 ± 2.889	45.846 ± 4.079
Squamous cell carcinoma (11 cases)	well	6	10.308 ± 2.729	182.63 ± 105.873	8.362 4.083	20.083 ± 7.198	59.717 ± 5.138	20.342 ± 2.17	28.902 ± 16.223
	moderately	4	10.393 ± 5.359	157.448 ± 110.379	9.938 ± 2.759	22.993 ± 3.081	52.825 ± 12.095	17.933 ± 4.852	29.693 ± 16.515
	poorly	1	9.26 ± 0.0	79.210 ± 0.0	7.710 ± 0.0	31.92 ± 0.0	51.20 ± 0.0	15.75 ± 0.0	46.93 ± 0.0
Large and small cell carcinoma (4 cases)	well	3	13.743 ± 4.787	165.083 ± 72.129	6.67 ± 2.327	23.003 ± 3.179	60.567 ± 3.356	20.46 ± 1.828	34.993 ± 6.582
	moderately	0	0	0	0	0	0	0	0
	poorly	1	14.45 ± 0.0	293.0 ± 0.0	13.64 ± 0.0	27.36 ± 0.0	72.4 ± 0.0	25.1 ± 0.0	38.69 ± 0.0

**Table 7:** Relationships between adenocarcinoma differentiations and parameters

Parameter	BV (p)	BF (p)	MTT (p)	PS (p)	PH (p)	PHpm/PHa (p)	MVD (p)
Well (n = 9) and moderately (n = 10)	0.679	1.000	0.198	0.999	0.738	0.615	0.945
Well (n = 9) and poorly (n = 5)	0.001	0.018	0.897	0.011	0.066	0.038	0.002
Poorly (n = 5) and moderately (n = 10)	0.003	0.025	0.130	0.038	0.035	0.014	0.002

*Because there are fewer cases of squamous cell carcinoma, large and small cell carcinoma, relationships between their differentiations and parameters were not compared. In the case of adenocarcinoma, BV, BF, PS, PHpm/PHa, and MVD between poorly and well differentiation and between poorly and moderately differentiation were statistically significant (all P < 0.05).

The peripheral lung cancers with VEGF positive expression showed significantly higher MVD value than that of peripheral lung cancers with VEGF negative expression (P < .05). MVD of benign nodules with VEGF positive and negative expression were not statistically significant (P > .05) (Table 8).

**Table 8:** Comparison among the perfusion parameters and MVD between VEGF positive and negative expression of three groups of peripheral pulmonary nodules

Pulmonary nodule	Parameter	BV	BF	MTT	PS	PH	PHpm/PHa (100%)	MVD
Peripheral lung cancer	VEGF positive expression (n = 29)	11.457 ± 3.593	225.406 ± 83.556	7.326 ± 3.807	21.638 ± 7.050	62.531 ± 8.579	21.285 ± 3.238	32.495 ± 14.541
	VEGF negative expression (n = 10)	8.025 ± 2.859	146.728 ± 88.744	7.896 ± 3.787	14.090 ± 6.785	55.740 ± 5.132	18.657 ± 2.466	20.002 ± 9.363
	F	7.449	6.394	0.167	8.680	5.522	5.455	6.400
	P	0.010	0.016	0.685	0.006	0.024	0.025	0.016
Inflammatory nodule	VEGF positive expression (n = 0)	/	/	/	/	/	/	/
	VEGF negative expression (n = 13)	8.542 ± 2.581	160.902 ± 68.412	6.397 ± 3.292	4.019 ± 1.730	65.700 ± 5.948	22.163 ± 2.240	33.966 ± 15.548
Benign nodule	VEGF positive expression (n = 3)	6.620 ± 2.231	83.070 ± 25.153	6.320 ± 1.890	5.047 ± 2.945	30.733 ± 14.199	9.793 ± 4.177	8.877 ± 3.285
	VEGF negative expression (n = 9)	5.534 ± 1.439	79.954 ± 13.919	5.573 ± 3.222	3.380 ± 1.903	26.867 ± 7.652	8.592 ± 2.168	7.653 ± 2.081
	F	1.000	0.078	0.139	1.350	0.386	0.448	0.599
	P	0.341	0.786	0.717	0.272	0.548	0.519	0.457

*Data is the mean ± SD.

For VEGF negative expression, MVD of inflammatory nodule were significantly higher than that of peripheral lung cancer (P < .05), MVD of benign nodule were significantly lower than that of peripheral lung cancer and inflammatory nodule (P < .05) (Tables 8, 9).

**Table 9:** Comparison among the perfusion parameters and MVD between VEGF positive and negative expression of three groups of peripheral pulmonary nodules

	Parameter		BV	BF	MTT	PS	PH	PHpm/PHa	MVD
VEGF positive expression	Peripheral lung cancer (n = 29) and benign nodule (n = 3)	P	0.031	0.007	0.658	0.000	0.000	0.000	0.010
VEGF negative expression	Peripheral lung cancer (n = 10) and inflammatory nod (n = 13)	P	0.616	0.968	0.308	0.003	0.000	0.001	0.043
	Peripheral lung cancer (n = 10) and benign nodule (n = 9)	P	0.033	0.114	0.152	0.002	0.000	0.000	0.007
	Inflammatory nodule (n = 13) and benign nodule (n = 9)	P	0.008	0.003	0.585	0.818	0.000	0.000	0.000

### 3. Perfusion parameters of pulmonary nodules

PH, PHpm/PHa, BF and BV values of peripheral lung cancer and inflammatory nodule were not statistically significant (all P > .05). Both showed significantly higher PH, PHpm/PHa, BF, BV value than those of benign nodule (all P < .05). MTT value was not statistically significant among the three groups of nodules (all P > .05). Peripheral lung cancer showed significantly higher PS value than that of inflammatory nodule and benign nodule (all P < .05). PS value of inflammatory nodule and benign nodule was not statistically significant (P > .05) (Tables 3, 4).

BV, BF, PS, MTT, PH, and PHpm/PHa among adenocarcinoma, squamous cell carcinoma, large and small cell carcinoma were not significantly (all P > 0.05) (Table 5). In the case of adenocarcinoma, BV, BF, PS, PHpm/PHa between poorly and well differentiation and between poorly and moderately differentiation were statistically significant (all P < 0.05) (Tables 6, 7).

The peripheral lung cancers with VEGF positive expression showed significantly higher PH, PHpm/PHa, BF, BV, and PS values than those of the peripheral lung cancers with VEGF negative expression, and than those of benign nodule with VEGF positive expression (Table 8).

In the case of VEGF negative expression, the PH, and PHpm/PHa of inflammatory nodule were significantly higher than those of peripheral lung cancer (all P < .05). The PS of inflammatory nodule was significantly lower than that of peripheral lung cancer (P < .05). Both BF, BV, and MTT were not statistically significant (all P > .05). PH, PHpm/PHa, BF, and BV of benign nodule were significantly lower than those of inflammatory nodule (all P < .05), rather than PS and MTT (all P > .05). PH, PHpm/PHa, BV, and PS of benign nodule were significantly lower than those of peripheral lung cancer (all P < .05), rather than BF, and MTT (all P > .05) (Tables 8, 9).

MVD, but not MTT, was positively correlated with PH, PHpm/PHa, BF, BV, and PS of the peripheral lung cancer with VEGF positive expression. MVD was positively correlated with PS of the benign nodule with VEGF positive expression, rather than PH, PHpm/PHa, BF, BV, and MTT. MVD was not correlated with PH, PHpm/PHa, BF, BV, PS, as well as MTT of the three groups of pulmonary nodules with VEGF negative expression (Table 10).

**Table 10:** Correlation between MVD and perfusion parameters of three groups of peripheral pulmonary nodules with VEGF positive and negative expression

Pulmonary nodule	Parameter	VEGF positive expression		VEGF negative expression	
			
		r	p	r	p
Peripheral lung cancer	BV	0.429	0.020	0.106	0.771
	BF	0.432	0.019	0.013	0.971
	MTT	-0.218	0.256	-0.174	0.630
	PS	0.512	0.004	0.114	0.754
	PH	0.444	0.016	0.533	0.112
	PHpm/PHa	0.415	0.025	0.452	0.190
Inflammatory nodule	BV	/	/	0.135	0.661
	BF	/	/	0.104	0.734
	MTT	/	/	-0.252	0.407
	PS	/	/	0.299	0.321
	PH	/	/	0.067	0.829
	PHpm/PHa	/	/	0.344	0.250
Benign nodule	BV	0.957	0.188	0.407	0.277
	BF	0.996	0.055	0.360	0.341
	MTT	-0.992	0.083	-0.544	0.130
	PS	0.999	0.026	0.535	0.138
	PH	0.991	0.086	0.441	0.235
	PHpm/PHa	0.983	0.116	0.468	0.203

### 4. Perfusion parametric maps of pulmonary nodules

There were 3 types of CT perfusion parametric maps of pulmonary nodules. Type I was lack of central blood perfusion with sufficient peripheral blood perfusion (Figures 2C–2F, 5C–5F, 6C–6F); Type II was heterogeneity blood perfusion and much less blood perfusion in the area of necrosis (Figures 3C–3F); Type III was lack of central and peripheral blood perfusion (Figures 4C–4F).

There were 21 patients in type I cases (13 peripheral lung cancers, 2 inflammatory nodules, 4 tuberculoms, 2 hamartomas); 33 patients in type II cases (24 peripheral lung cancers, 9 inflammatory nodules) and 10 patients in type III cases (2 peripheral lung cancers, 2 inflammatory, 5 tuberculomas, 1 hamartoma). The perfusion pattern of peripheral lung cancer and inflammatory nodule was not statistically significant (F = 2.559, P = 0.298). Both and benign nodule were statistically significant (F = 19.984, P = 0.000; F = 13.562, P = 0.001) (Table 11).

**Table 11:** Type of perfusion parametric maps showing three groups of pulmonary nodules

	TypeI	TypeII	Type III
Peripheral lung cancer(n = 39)	13	24	2
Inflammatory nodule(n = 13)	2	9	2
Benign nodule(n = 12)	6	0	6

*The perfusion patterns of peripheral lung cancer and inflammatory nodule were not statistically significant (F = 2.559, p = 0.298), but both and benign nodule were statistically significant (F = 19.984, P = 0.000; F = 13.562, P = 0.001).

The size of the tumor in CT perfusion parametric maps was smaller than that of CT original images and there were more exudative process found in the nodules (Figures 4B–4F).

## Discussion

A growing tumor needs added blood supply from adjacent tissues, because this is essentially required for tumor growth and metastatic spread. This process may be caused by the increasing release of angiogenic factors from the tumor, such as vascular endothelial growth factor (VEGF), and subsequent increase in the extent of microvessel density (MVD) [11-14]. The resulting increase of MVD leads to increased perfusion and permeability of the blood capillaries. Owing to the increased MVD which is associated frequently with increased parameter values of a tumor at CT perfusion imaging [15], CT perfusion imaging can be interpreted as reflecting tumor vascularity. This discovery may aid in comparing the differentiation of benignancy and malignancy of the tumor [16,17].

### 1. Application value of TDC in the differentiation of benignancy and malignancy of the tumor

We want to look into early changes in the TDC in a region of interest which correlates with blood flow per unit of tissue. It is found that sufficient vascular characterization may be completely provided in the first scans after an intravenous bolus of contrast medium. CT perfusion imaging should be carried out during an early and first-pass process of contrast medium enhancement. Therefore, the data acquisition time for scanning is crucial. This study indicated that more intact TDC of all the nodules appeared within 40 seconds, and 2-second duration time after an intravenous bolus of contrast medium was carried out.

The different appearances of TDC of pulmonary nodules mainly represented the differences in blood perfusion and diffusion [18]. This study indicated that TDC of peripheral lung cancer had a steeper slope, and there was an obvious increase of nodular HU value. This changed little after reaching peak. In fact, it became flat at the peak, and had a platform. The enhancement of peripheral lung cancer and aorta enhanced at an equal pace. However, the peak of TDC appeared slightly later than that of the aorta. This was in accordance to the reports of Littleton [19] and Han Mingjun [20] who proposed that most lung cancers had abundant vascular supply, which was from the bronchial arterial system. The blood perfusion of peripheral lung cancer is abundant, so, there was a steeper slope in TDC.

The interstitial space of peripheral lung cancer was found to be larger, and a near absence or substantial reduction of lymphatic flow was noted. The retarded flow found in intravascular and interstitial spaces in the washout phase from the interstitial space contribute to retention of contrast medium in peripheral lung cancer. Thus, TDC of pulmonary nodule hanged out a little after reaching the peak. It was found to be flat at peak, with a platform.

This study indicated that TDC of inflammatory nodule had a steeper slope, which declined immediately after reaching the peak. It also indicated that in most of the processes of pulmonary inflammatory nodules, the number and sizes of bronchial arteries and blood circumfluence of bronchial veins had increased. Moreover, diffused thrombosis at the arterioles of the pulmonary circulation was observed and the microvessel structure was found to be made up of coarctate capillary network from the bronchial artery [19]. Moreover, it was found that the lung has two systems-bronchial artery and pulmonary artery of blood supply. When inflammatory nodules, ratio of ventilation and blood flow changed, the pulmonary arteries became narrow or occlusive, and bronchial artery became expanded. The vascular supply was actually found to be from the bronchial arteries. Thus, blood perfusion of the inflammatory nodule is abundant, and has a steeper slope in TDC. Flow of contrast medium through the intravascular space occurred through relatively straight vessels with a normal configuration. There was an accelerated, active lymphatic flow, and diffusion of contrast medium increased. Thus, TDC of inflammatory nodule declined earlier than that of peripheral lung cancer after reaching the peak.

This study indicated that TDC of benign nodule (tuberculoma and hamartoma) manifested as a flat trend. There was no a steeper slope, or an obvious increase in nodular CT value. Tuberculoma occurred mainly from caseous material and hamartoma was mainly from cartilage and fibrous tissues. Therefore, MVD is smaller, and vascular supply of tuberculoma and hamartoma is not abundant. There is lesser blood circumfluence in vessels and extravascular space. This resulted in a faster diffusion in tuberculoma and hamartoma. Thus, TDC of benign nodule manifested in a flat trend. Tthere was no steeper slope, or increase in nodular CT value.

This study indicated that TDC may depict a hemadynamic character of the pulmonary nodules. These characterizations may act as reference indexes for differentiating benign and malignant pulmonary nodules. TDC of peripheral lung cancer was similar to that of inflammatory nodule. Both TDC were shown to be obviously different from that of benign nodule. This coincided with the study of Zhang et al. [21]. However, Zhang et al. [21] believed that after inflammatory nodules reached maximum enhancement, the TDC declined and then rose again. This did not coincide with our study for two reasons. Firstly, contrast medium distributed in the vessels rather than in extravascular space during data acquisition of 40-seconds. After an intravenous bolus of conventional iodinated contrast material, the TDC reflected sufficient vascular characterization but did not reflect TDC of extravascular characterization in this study. Secondly, this discovery correlated with the scanner mode and application of the 16-slice spiral CT machine in this study.

TDC of benign nodule was obviously different from that of peripheral lung cancer and inflammatory nodule. When TDC manifested as a flat trend, there is neither a steeper slope nor obvious increase of nodular HU value. Therefore, morphologic characteristics and patient history may be used, and tuberculoma or hamartoma may be considered first before other pulmonary lesions are proved.

### 2. Clinical application value of perfusion parameters in the differentiation of benignancy and malignancy of tumor

This study indicated that PH, PHpm/PHa, BF, BV value of peripheral lung cancer and inflammatory nodule were not statistically significant. Both showed significantly higher PH, PHpm/PHa, BF, BV value than those of benign nodule. Peripheral lung cancer showed significantly higher PS value than that of inflammatory nodule and benign nodule. PS value of inflammatory nodule and benign nodule were not statistically significant. MTT value was not statistically significant among the three groups of nodules. In the case of adenocarcinoma, BV, BF, PS, PHpm/PHa between poorly and well differentiation and between poorly and moderately differentiation were statistically significant.

The reason may be owing to the fact that the vascular supply of malignant nodule and inflammatory nodule is more abundant, and therefore, both blood perfusion have increased. The vascular supply of benign nodule is lesser, therefore, its blood perfusion is obviously less than that of malignant nodule and inflammatory nodule. This coincided with the study results of Swensen et al. [5,7,22,23] and Zhang et al. [21], who compared PH, PHpm/PHa or perfusion value of malignant, inflammatory, and benign nodule, and suggested that malignant and inflammatory nodule showed higher PH, PHpm/PHa or higher perfusion value than those of benign nodule.

The result of this study indicated that there was a better consistency among BF, BV, PS, PH, PHpm/PHa in peripheral lung cancer, inflammatory nodule, and benign nodule cases. This result suggested that the five perfusion parameters may better reflect blood perfusion status, malignant and benign degree of pulmonary nodules.

PS value reflects one-way transmission speed for contrast material to enter cell spaces by capillary endothelium, and it reflects the diffusion coefficient of blood vessel endothelial interspace of tumor angiogenesis. There were straighter branches of blood vessels, mature blood capillaries, and near normal permeability surfaces of blood capillaries in benign and inflammatory nodules. Therefore, PS value of inflammatory nodule and benign nodule was lower, and not statistically significant. For peripheral lung cancer, there is found to have more developmental immaturity of tumor capillary, no intact vessel wall, and increased permeability surface of blood capillary which resulted from VEGF and other vascular growth factors. Therefore, the PS value of peripheral lung cancer is higher.

PH, PHpm/PHa, BF, BV value of peripheral lung cancer and inflammatory nodule were usually significantly higher than those of benign nodule. Peripheral lung cancer usually showed significantly higher PS value than that of inflammatory nodule and benign nodule. when a pulmonary nodule shows that PH, PHpm/PHa, BV and BF are higher, PS is usually higher. It is possible that before other pulmonary nodules are found, peripheral lung cancer may be considered first. When pulmonary nodules show PH, PHpm/PHa, BV, BF are higher, PS is found to be lower, and inflammatory nodule may be considered first.

### 3. Application value of perfusion parametric maps of pulmonary nodules

The findings of our study indicated that perfusion map in type I usually showed up in tuberculoma and hamartoma, and there was shown to be a lack of central blood perfusion, and sufficient peripheral blood perfusion. Perfusion map in type II usually showed up in inflammatory nodule and peripheral lung cancer. In this case, there was the presence of heterogeneity blood perfusion, and area of necrosis was found to be less. Perfusion map in type III usually also showed up in tuberculoma, hamartoma and inflammatory nodule, and there was found to be a lack of central and peripheral blood perfusion.

In perfusion parametric map of pulmonary nodule, there is found to be a lack of central blood perfusion and sufficient peripheral blood perfusion before other pulmonary lesions are proved. Morphologic characteristics and patient history may be used, and benign pulmonary nodule (tuberculoma or hamartoma) may be considered first. However, other pulmonary nodules may found not to completely excluded, because some adenocarcinomas may be filled with mucus. Also fewer inflammatory nodules may be filled with pus, and have lack of central perfusion.

This study indicated that the low-perfusion area of necrosis and mucus or cavitation area could diminish the mean perfusion parameter values. Therefore, when drawing up region of interest, it is considered that pulmonary nodule, low perfusion and cavitation should be avoided as far as possible.

The size of tumor in CT perfusion parametric maps was usually smaller than that of original images when there were more exudative process shown in tumors. Therefore, CT perfusion parametric maps displayed by color gray scale may reveal the essence of pulmonary nodules and differences in regional blood perfusion status more directly, clearly, realistically, and reliably. This will probably aid in paracentesis and biopsy of the essence of pulmonary nodule by CT guidance.

### 4. Relationship between perfusion parameters and MVD and VEGF in pulmonary nodules

Angiogenesis is a complicated process, as it is controlled by vascular growth factors and vascular growth inhibitor factors [24,25], This is because the endothelial cells which have resided in blood capillary and postcapillary vein will enter again itto cell generation cycle. As a result, there will be degrading basal membrane under endothelia cells and this will form blood capillary spore. In turn, blood capillary will move towards peripheral tissues by a pullulate way. Thus, when a tumor has no passage to directly contact with the eutaxia circulation system, it must induce the vicinity vessels to erupt the new vessels.

Growth of tumor will suffer limitation if there is occurrence of no angiogenesis. Tumor cells obtain nutrition only when there is diffusion. Tumor will stop growing after achieving 1–2 nm diameter or thickness of about 10^7 ^cells if there is not enough oxygen and nutrition supply. However, growth of tumor will accelerate once new vessels are put into tumor tissues, and the nutrition supply of tumor transforms perfusion from diffusion process. Therefore, the tumor is a growth that typically depends on supply from the blood vessels. Although there are many kinds of vascular growth factors, VEGF played a crucial role in tumor angiogenesis because of its speciality factor in promoting tumor karyokinesis [25]. MVD is the number of microvessel per unit area which is counted by using some specific antibodies (for instance CD34 monoclonal antibody)to mark microvessel endothelial cells of tumor tissues.

This study indicated that in the case of VEGF positive expression, MVD was found to be positively correlated with PH, PHpm/PHa, BF, BV, and PS of peripheral lung cancer. This coincided with the studies of Yi et al. [26], Tateishi et al. [27] and Li Shenjiang et al. [8]. Yi et al. and Tateishi et al. described that a significantly positive correlation was found between enhancement of MVD and/or VEGF of malignant pulmonary nodule. Li Shenjiang et al [8]. reported that enhancement and perfusion value were significantly and positively correlated with MVD and VEGF of malignant pulmonary nodule, and MTT value was not significantly correlated with MVD and VEGF.

This study also indicated that VEGF expression of 32 cases (29 peripheral lung cancer, 0 inflammatory nodule, 3 benign nodule) were positive. MVD was not correlated with PH, PHpm/PHa, BF, BV, and PS of three groups of pulmonary nodules with VEGF negative expression. The peripheral lung cancers with VEGF positive expression showed significantly higher PH, PHpm/PHa, BF, BV, and PS value than those of the peripheral lung cancers with VEGF negative expression, and those of benign nodule with VEGF positive expression.

It is clear that PH, PHpm/PHa, BF, BV, and PS value can reflect MVD measurement and VEGF expression of benign and malignant pulmonary nodules. This explains that PH, PHa/PHpm, BF, BV, PS may act as angiogenesis index of pulmonary nodules, and have potential value of studied pulmonary nodule angiogenesis.

## Conclusion

16-slice spiral CT perfusion imaging closely correlated with tumor angiogenesis and reflected MVD measurement and VEGF expression. It provided not only a non-invasive method of quantitative assessment for blood flow patterns of peripheral pulmonary nodules.

## Abbreviations

VEGF: Vascular endothelial growth factor; TDC: Time density curve; MVD: Microvessel density; PH: Peak height; PHpm/PHa: Peak height ratio of pulmonary nodule to aorta; BF: Blood flow; BV: Blood volume; PS: Permeability surface; MTT: Mean transit time.

## Competing interests

The authors declare that they have no competing interests.

## Authors' contributions

SHM created the original study design, wrote the protocol, and extracted the data. HBL, BHJ and ZXW read an earlier version of the manuscript and gave insightful comments and suggestions. SHM, HBL and XLC undertook CT scanning and analyses of CT images. SHM, HBL and WM inquired about cases and undertook immunohistochemical staining. ZXW and ZGH did statistical testings. All authors contributed to both protocol and final reports.

## Pre-publication history

The pre-publication history for this paper can be accessed here:


